# Application of Priming Strategy for Enhanced Paclitaxel Biosynthesis in *Taxus* × *Media* Hairy Root Cultures

**DOI:** 10.3390/cells11132062

**Published:** 2022-06-29

**Authors:** Katarzyna Sykłowska-Baranek, Grażyna Sygitowicz, Agata Maciejak-Jastrzębska, Agnieszka Pietrosiuk, Anna Szakiel

**Affiliations:** 1 Department of Pharmaceutical Biology and Medicinal Plant Biotechnology, Faculty of Pharmacy, Medical University of Warsaw, 1 Banacha Str., 02-097 Warsaw, Poland; katarzyna.syklowska-baranek@wum.edu.pl (K.S.-B.); agnieszka.pietrosiuk@wum.edu.pl (A.P.); 2 Department of Clinical Chemistry and Laboratory Diagnostics, Medical University of Warsaw, 1 Banacha Str., 02-097 Warsaw, Poland; agata.maciejak@wum.edu.pl; 3 Department of Plant Biochemistry, Faculty of Biology, University of Warsaw, 1 Miecznikowa Str., 02-096 Warsaw, Poland; szakal@biol.uw.edu.pl

**Keywords:** β-aminobutyric acid, elicitation, gene expression, methyl jasmonate, sodium nitroprusside, L-phenylalanine

## Abstract

Despite huge progress in biotechnological approaches to paclitaxel production, *Taxus* spp. in vitro culture productivity still remains a challenge. This could be solved by developing a new strategy engaging mechanisms of the primed defence response joined with subsequent elicitation treatment to circumvent limitations in paclitaxel biosynthesis. The hairy roots were primed by preincubation with β-aminobutyric acid (BABA) for 24 h or 1 week, and then elicited with methyl jasmonate (MeJA) or a mixture of MeJA, sodium nitroprusside and L-phenylalanine (MIX). The effect of priming was evaluated on a molecular level by examination of the expression profiles of the four genes involved in paclitaxel biosynthesis, i.e., *TXS* (taxadiene synthase), *BAPT* (baccatin III: 3-amino, 3-phenylpropanoyltransferase), *DBTNBT* (3′-*N*-debenzoyl-2-deoxytaxol-*N*-benzoyltransferase) and *PAM* (phenylalanine aminomutase), as well as *rolC* (cytokinin-β-glucosidase), originated from the T-DNA of *Agrobacterium rhizogenes*. The maximum paclitaxel yield was achieved in cultures primed with BABA for 1 week and elicited with MIX (3179.9 ± 212 µg/g dry weight), which corresponded to the highest expression levels of *TXS* and *BAPT* genes. Although BABA itself induced the investigated gene expression over control level, it was not translated into paclitaxel production. Nevertheless, preincubation with BABA essentially affected paclitaxel yield, and the duration of BABA pretreatment seemed to have the most pronounced impact on its productivity.

## 1. Introduction

Since its discovery in the 1960s, paclitaxel has become a well-established and extensively used anticancer drug in the treatment of a wide variety of malignancies, including breast and ovarian cancers, non-small-cell lung cancer, head and neck tumours, Kaposi’s sarcoma and urologic cancers [1,2]. Paclitaxel is also considered as a chemotherapeutic in Alzheimer’s disease [3], as well as in restenosis treatment [4]. The development of numerous new practical applications of paclitaxel continues to place this molecule in the centre of scientific interest, covering not only its chemistry and novel formulations, but also the aspects of paclitaxel supply. Although paclitaxel is now produced semisynthetically from its analogues, biotechnological methods for its production on a commercial scale have also been elaborated, and the FDA (Food and Drug Administration, USA) has approved plant cell cultures as a renewable source of paclitaxel [5]. Nevertheless, despite huge progress in biotechnological approaches to paclitaxel production, *Taxus* spp. cell culture productivity still remains a challenge. This could be solved by the identification of the limiting steps at the molecular level, and the elaboration of a new strategy involving the primed defence response approach combined with subsequent elicitor treatment to circumvent limitations in paclitaxel biosynthesis.

Although intensively studied, the taxane biosynthetic pathway is not fully characterised. According to the current knowledge, it was revealed that it requires approximately 20 enzymatic steps [5]. Further, the possible rate-limiting steps in taxane biosynthesis were indicated to be controlled by genes encoding enzymes of the late pathway: baccatin III: 3-amino, 3-phenyl-propanoyltransferase (*BAPT*), the enzyme which integrates baccatin III with phenylisoserine to 3′-*N*-debenzoyl-2-deoxytaxol, and 3′-*N*-debenzoyl-2-deoxytaxol-*N*-benzoyltransferase (*DBTNBT*), the enzyme which produces 2′-deoxytaxol through ligation of benzoyl CoA and 3′-*N*-debenzoyl-2-deoxytaxol.

Being constantly exposed to various biotic and abiotic stressors, plants evolved innate immunity systems which are composed of structural barriers and inducible defence responses, including defence gene expression and phytoalexin production, as well as constitutive secondary metabolite accumulation, to cope with pathogen attacks [6,7,8]. The recognition of certain signals from their environment (microbes, pathogens, abiotic stress, chemical compounds) results in cells trigging to the primed state of long-lasting and enhanced defence, both in affected and untreated (naïve) parts of the plant. Once primed, plants are capable of exerting faster and more pronounced responses to different stressors.

β-aminobutyric acid (BABA), a non-protein amino acid, has been proved to be an effective inducer of resistance in many plant species via priming [8,9]. BABA was reported to potentiate secondary metabolite production when subjected to subsequent elicitor treatment, which was reviewed in detail by Cohen [10]. Nevertheless, investigations on BABA application to in vitro plant cell cultures to potentiate secondary metabolism are limited. Further, up to now there is one report on BABA application to hairy root cultures of *Salvia militiorrhiza* [11]. The influence of preincubation with BABA (0.1–2 mM) or methyl jasmonate (MeJA; 5–200 µM) for different time periods (0–3 days) on root response to fungal elicitor (yeast extract, YE) was examined. It was demonstrated that pretreatment with BABA for 3 days before YE addition resulted in the highest tanshinone content. Moreover, when BABA and YE were applied simultaneously, the yield of tanshinones was on a parallel level with the sole presence of YE. In addition, in pretreated BABA cultures, the steady rise in tanshinone content was noted over the whole pretreatment time. The effect of MeJA preincubation was similar to that of BABA, but less pronounced; however, BABA suppressed hairy root growth to a higher extent than MeJA.

Although BABA was reported to be an effective inducer of defence responses in a wide range of plant species [10], mono- and dicots, nothing is known about its mode of action in gymnospermous plants.

The aim of the present study was to induce the primed response by application of β-aminobutyric acid, followed by elicitor treatment to enhance paclitaxel production in hairy root cultures of *Taxus* × *media*. Furthermore, an effort to elucidate the molecular basis for observed cell response was undertaken. The priming strategy was applied for the improvement of paclitaxel production in *Taxus* spp. in vitro cultures for the first time.

## 2. Materials and Methods

### 2.1. Reagents

All reagents used to fulfil the aims of this study were purchased from Sigma-Aldrich (Poznań, Poland) or from Avantor Performance Materials Poland S.A. (Gliwice, Poland). The standard compound of paclitaxel was produced by ChromaDex (Los Angeles, CA, USA), and purchased from LCG Standards (Teddington, Middlesex, UK).

### 2.2. Hairy Root Cultures and Treatment

The KT hairy root line resulting from agroinfection with *Agrobacterium rhizogenes* LBA 9402 [12] was subjected to investigation. Hairy roots were cultivated in 250 mL Erlenmeyer flasks containing 35 mL of modified hormone-free liquid DCR medium [13] and routinely subcultured every four weeks. The cultures were performed on a gyratory shaker at 105 rpm (TR 250 INFROS AG, Bottingen, Switzerland) in the dark at 23 ± 1 °C.

Twenty-eight-day-old roots were subjected to experiments. The following culture variants were carried out: (i) control culture—untreated 28-day-old roots (day ”0”); (ii) treated with ETOH 35 µL per flask; (iii) elicited with 100 µM methyl jasmonate (MeJA); (iv) elicited with 100 µM MeJA, 100 µM L-phenylalanine and 10 µM sodium nitroprusside (all together denoted as MIX); (v) primed with 100 µM β-aminobutyric acid (BABA) for 1 h, 3 h, 6 h, 12 h, 18 h, 24 h, 48 h, 72 h, 5 days, 7 days and 14 days; (vi) primed 24 h with 100 µM BABA and subsequently supplemented with ETOH 35 µL per flask (BABA24E); (vii) primed 24 h with 100 µM BABA and subsequently elicited with 100 µM MeJA (BABA24MeJA); (viii) primed 24 h with 100 µM BABA and subsequently elicited with MIX (BABA24MIX); (ix) primed 1 week with 100 µM BABA and subsequently supplemented with ETOH 35 µL (BABA1E); (x) primed 1 week with 100 µM BABA and subsequently elicited with 100 µM MeJA (BABA1MeJA); (xi) primed 1 week with 100 µM BABA and subsequently elicited with MIX (BABA1MIX). For phytochemical analysis, the samples were collected after 24 h, 48 h, 72 h, 7 days and 14 days.

The hairy root growth in control culture was determined based on fresh weight (FW) increase using the following equation [14]:

FW_28d_ = m_28d_ × m_0d_^−1^ [−] (1)

where m_28d_ is the FW of hairy roots after 28 days of culture (FW_28d_), m_0d_ is FW at day of inoculation. Meanwhile, hairy root growth in treated cultures was determined based on the equation:

FW_14d_ = m_(14d)_ × m_0d_^−1^ [−] (2)

where m_(14d)_ is the FW of hairy roots after 14 days of treatment (FW_14d_), m_0d_ is FW at day of inoculation (day “0”).

### 2.3. Quantitative Real-Time PCR (qRT-PCR)

The expression profiles of the four genes involved in paclitaxel biosynthesis, i.e., *TXS*, *BAPT*, *DBTNBT* and *PAM*, as well as *rolC* (originated from T-DNA of *A*. *rhizogenes)*, were investigated. *TXS* commits first step of paclitaxel and other taxane biosynthesis, i.e., converts geranylgeranyl pyrophosphate to taxa-4(5), 11(12)-diene; *PAM* plays role in formation of β-phenylalanoyl-CoA side chain from L-phenylalanine, while *BAPT* and *DBTNBT* are engaged in late-pathway taxane biosynthesis steps. *BAPT* catalyses conjugation of the β-phenylalanoyl-CoA side chain to the C13 hydroxyl group of baccatin III to generate 3′-*N*-debenzoyl-2-deoxytaxol. *DBTNBT* participates in formation of 2′-deoxytaxol through ligation of benzoyl CoA group to 3′-*N*-debenzoyl-2-deoxytaxol [15,16]. For gene expression analysis, primed BABA24 or BABA1 and elicited culture samples were collected after 1 h, 3 h, 6 h, 12 h, 18 h, 24 h, 48 h, 72 h. The total RNA was isolated from hairy roots frozen in liquid nitrogen according to the method described by Sykłowska-Baranek et al. [17]. One µ of total RNA was used to reverse transcription carried out with cDNA RevertAid First Strand cDNA Synthesis Kit and random hexamer primer (Thermofisher Scientific, Waltham, MA, USA) according to the manufacturer’s instruction.

Quantitative real-time PCR (qRT-PCR) was used to analyse baccatin III: 3-amino, 3-phenylpropanoyltransferase (*BAPT*), 3′-*N*-debenzoyl-2-deoxytaxol-N-benzoyltransferase (*DBTNBT*), phenylalanine aminomutase (*PAM*), cytokinin-beta-glucosidase (*rolC*), taxadiene synthase (*TXS*) gene expression changes in investigated *T*. × *media* hairy roots during elicitation. The primer sequences were designed using Primer3, Version 0.4.0. Verification of primers’ matching and specificity was performed by in silico validation, using Basic Local Alignment Search Tool (BLAST). The sequences of the primers are presented in Supplementary Table S1. The qPCR reactions for *BAPT*, *DBTNBT*, *PAM*, *TXS* genes were performed using 2X PowerUp™ SYBR™ Green Master Mix (Applied Biosystems by Life Technologies, Austin, TX, USA), whereas for *rolC* gene using 2X SYBR™ Green PCR Master Mix (Applied Biosystems by Life Technologies, Austin, TX, USA) in 10 μL final reaction volume according to the manufacturer’s protocols. The cDNA templates were 20-fold diluted and the final concentration for each primer was 500 nM. Each qPCR reaction was performed in duplicate on 96-well FrameStar plates (4titude^®^ Ltd., Wotton, UK) using the LightCycler^®^ 480 II instrument (Roche Diagnostics GmbH, Mannheim, Germany). The temperature cycling conditions were for 2X PowerUp™ SYBR™ Green Master Mix as follow: UDG activation at 50 °C for 2 min, Dual-Lock™ DNA polymerase at 95 °C for 2 min, followed by 40 cycles of amplification at 95 °C for 15 s, 60 °C for 15 s and 72 °C for 1 min; whereas for 2X SYBR™ Green PCR Master Mix: AmpliTaq Gold^®^ polymerase activation at 95 °C for 10 min followed by 40 cycles of amplification at 95 °C for 15 s and 60 °C for 1 min. Specificity of the primer amplicons was confirmed by melting curve analysis and 2% agarose gel electrophoresis. No-template controls (NTCs) were included in every qPCR run. Raw qPCR data were analysed using LightCycler 480 Software, Version 1.5.0 SP3. Number of quantification cycles were determined using the second derivative maximum method. The qPCR data of the candidate reference genes *18S rRNA*, 3,5-epimerase-4-reductase-like protein (*TBC41*), β-tubulin (*TUBB*) were analysed using the tool RefFinder (https://heartcure.com.au; accessed on 14 October 2020), available online. Based on the rankings from geNorm (Supplementary Figure S1), final gene expression data were normalised to the most stable reference genes *TUBB* and *TBC41*, the latter was also chosen as a reference gene in study by Sabater-Jara et al. [18]. Fold change of the gene expression, PCR efficiency-corrected, was calculated using REST 2009 Relative Expression Software Tool, Version 2.0.13 (Qiagen GmbH, Hilden, Germany).

### 2.4. Paclitaxel Quantitative Determination Using HPLC-UV-DAD Method

Paclitaxel concentration was determined in hairy roots cultivated in treated cultures and compared to its content in control. At set time points, roots were collected and gently pressed on the filter paper and lyophilised (Christ ALPHA 1-4 LSC; Osterode am Harz, Germany). Afterwards, roots were powdered (100 mg) and extracted with 1 mL MEOH by 15 min sonication (Sonorex; Bandelin, Berlin, Germany), and left overnight on gyratory shaker in the dark. Next, samples were centrifugated at 15,500× *g* (EBA 12R Hettich, Tuttlingen, Germany), extracts were transferred to fresh Eppendorf tubes and samples were re-extracted with 1 mL MEOH. Finally, extracts were collected, evaporated and cleaned using SPE method developed by Theodoridis [19]. Paclitaxel determination was performed according to Sykłowska-Baranek et. al. [20], applying the method described by Theodoridis [21].

All experiments were carried out in triplicate and the statistical significance between means was assessed by the Kruskal–Wallis one-way analysis of variance using STATISTICA 13.1 PL software (StatSoft Polska; Kraków, Poland). A probability of *p* < 0.05 was considered as significant. Pair-wise correlations were calculated by Pearson’s correlation coefficient test.

## 3. Results

### 3.1. Hairy Root Cultures and Paclitaxel Biosynthesis

**Figure 1:**
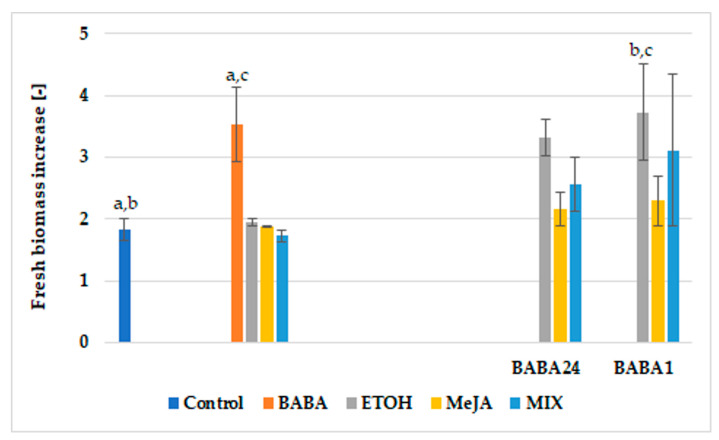
Fresh biomass increase of hairy roots cultivated under various culture conditions: control—untreated roots collected at day of inoculation (day “0”); BABA—roots treated with 100 µM β-aminobutyric acid (BABA) for 14 days; ETOH—roots treated with ETOH for 14 days without BABA, and variants: treated 24 h or 1 week with BABA with subsequent addition of ETOH for 14 days; MeJA—roots treated with MeJA for 14 days without BABA, other variants: treated 24 h or 1 week with BABA with prior to addition of MeJA for 14 days; MIX—roots cultivated for 14 days without BABA, and variants: treated 24 h or 1 week with BABA with subsequent addition of MIX of elicitors. Data represent mean values ± SD from three independent experiments. Data within groups denoted with the same letters are statistically significant (*p* ˂ 0.05).

The primed defence response approach, combined with subsequent elicitor treatment, was applied in the current study to enhance paclitaxel biosynthesis in hairy root cultures of *T*. × *media*. The examination of culture conditions on the biomass increase of the KT hairy root line showed that BABA applied alone significantly and almost 2-fold stimulated root growth in comparison to control (Figure 1). Additionally, in the BABA1E variant, root growth was over two times higher than in the control. Among elicited variants, this supplemented with MIX resulted in a slight growth decrease, although not statistically significant (*p* < 0.05).

**Figure 2:**
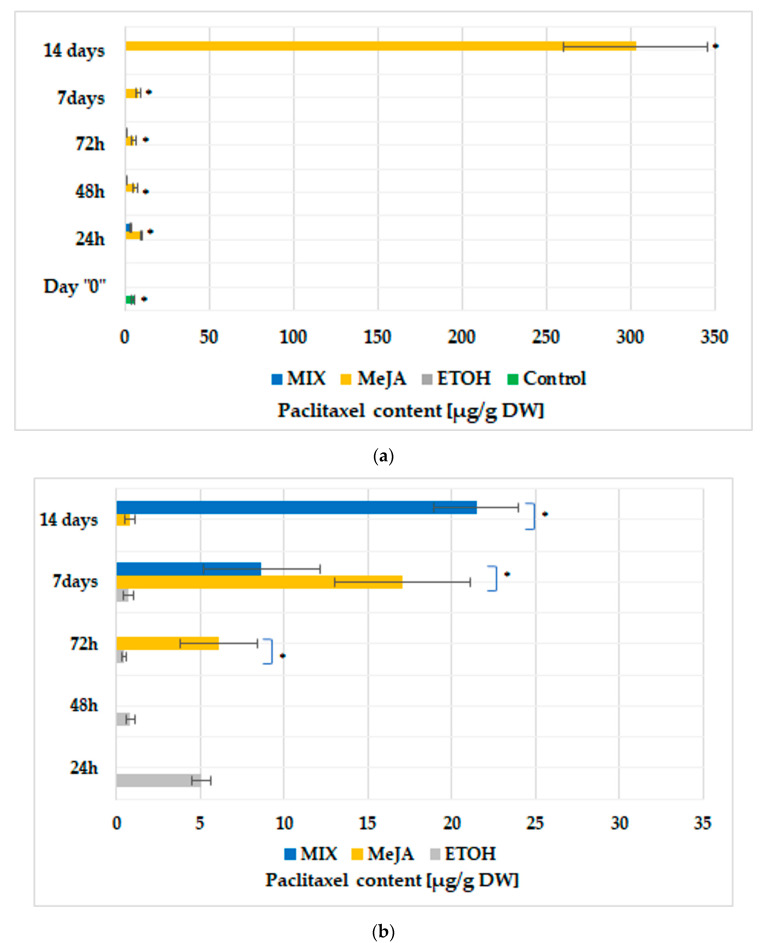

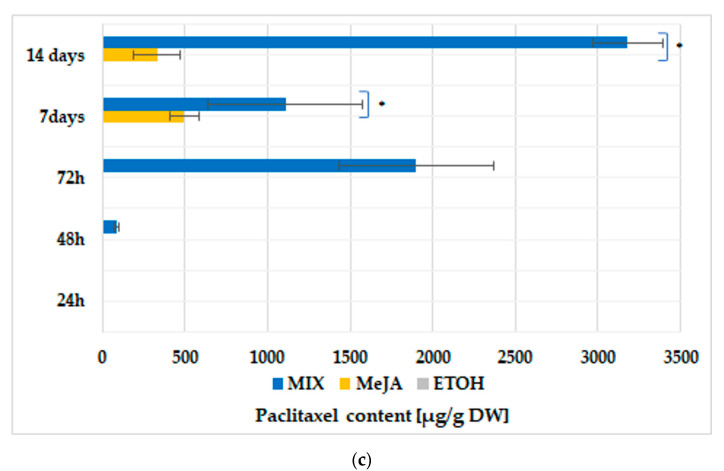
Paclitaxel content [µg/g DW] determined in hairy roots cultivated in various culture conditions: (**a**) without BABA supplementation; (**b**) elicited after treatment with BABA for 24 h; (**c**) elicited after treatment with BABA for 1 week. Data represent mean values ± SD from three independent experiments. Data denoted with asterisks (*) are statistically significant (*p* ˂ 0.05).

In culture variants without BABA, the highest paclitaxel content was determined after a 14-day-long elicitation with MeJA (303.1 ± 42.5 µg/g dry weight, DW), which was over 58-fold more than in the control (5.18 ± 1.7 µg/g DW) (Figure 2a). While under treatment with MIX, paclitaxel was detected only up to 72 h of culture, with its highest content noted after 24 h of elicitation. Nevertheless, it was a 2.4-fold lower concentration than after MeJA elicitation at the same time point (Figure 2a). The BABA applied alone, irrespective of duration of treatment, had no effect on paclitaxel biosynthesis. Medium supplementation with only ETOH did not induce paclitaxel production, in contrast to its application after 24 h of BABA treatment, where paclitaxel production was observed. Under those conditions, its highest yield was noted after 24 h, and decreased subsequently (Figure 2b). Among other BABA24 variants, BABA24MIX was the most favourable for paclitaxel accumulation, although its presence was detected for the first time on the 7th day of treatment, while its highest content amounted to 21.5 ± 2.5 µg/g DW, noted on day 14. In the BABA24MeJA variant, paclitaxel biosynthesis started after 72 h of culture and its highest yield (17.1 ± 4.0 µg/g DW) was achieved on day 7, which was then followed by a significant decline in its concentration. In the BABA1 variants, paclitaxel biosynthesis was determined only after elicitor/s addition. In the BABA1MeJA cultures, paclitaxel content was detected for the first time after 7 days of elicitation. At the same time point, it reached its maximum concentration of 495.7 ± 86.3 µg/g DW. In the BABA1MIX variant, paclitaxel accumulation started after 72 h of culture and rose up gradually up to 3179.9 ± 211.9 µg/g DW on day 14. It was the highest paclitaxel content determined under the conditions of the current study, over 613-fold higher than in control and over 6-fold higher than in the BABA1MeJA variant at its peak (495.7 ± 86.3 µg/g DW) (Figure 2c).

### 3.2. Gene Expression Profiles

**Figure 3:**
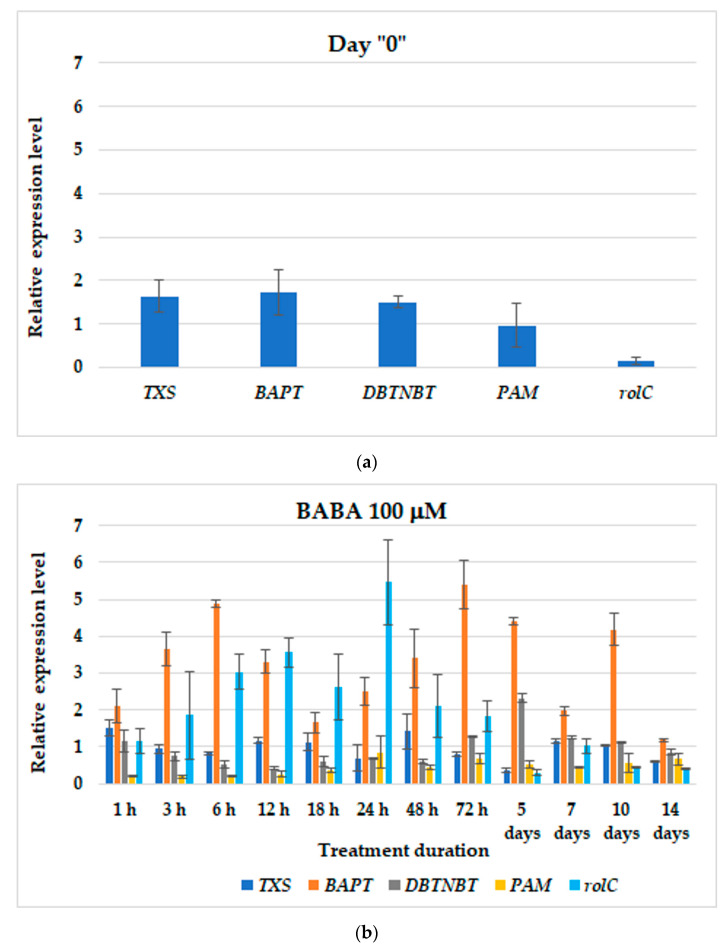
The gene expression profiles in control (**a**) and in 100 µM BABA-treated (**b**) hairy roots. Data represent mean values ± SD from three independent experiments.

In the control culture, the expression of early- as well as late-pathway genes was at a parallel level, with the lowest expression showed by *rolC* (Figure 3a). BABA affected the levels of *BAPT* and *rolC* genes the most (Figure 3b), however, they did not follow the same expression pattern, and, in addition, the correlation between them was negative (r = −0.01). The highest *BAPT* transcript level was noted 72 h after BABA addition, while for *rolC* it was after 24 h. For further experiments with BABA, two time points were chosen for elicitor supplementation, i.e., 24 h and 1 week. Relative expression levels of genes of interest between these time points did not differ significantly (*p* < 0.05), and they were also not significantly different than at other examined time points.

**Figure 4:**
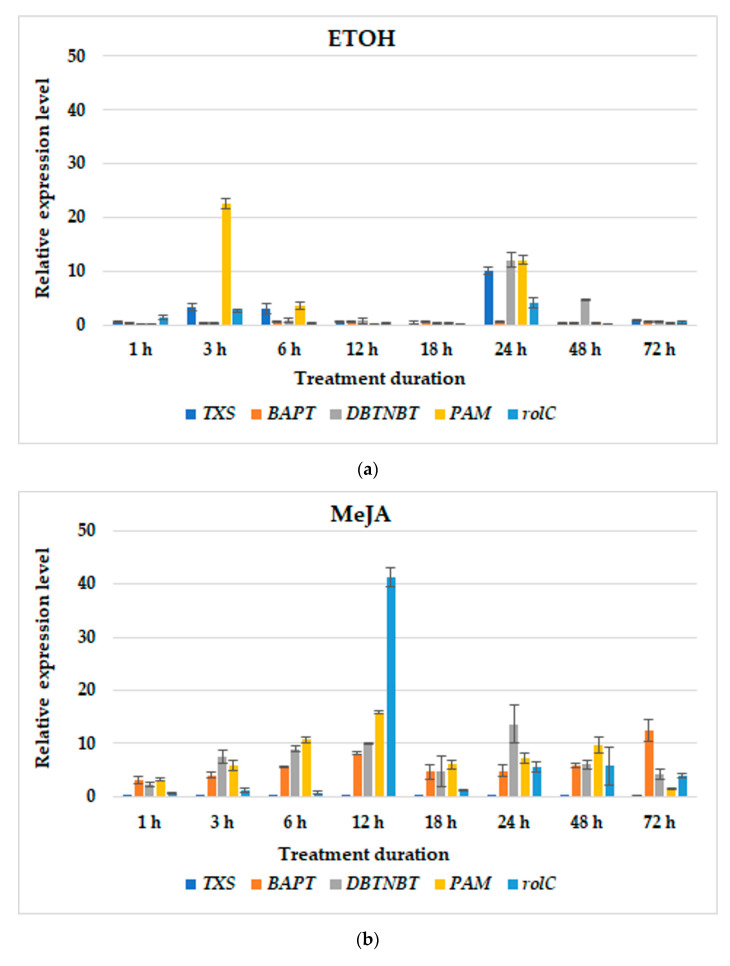

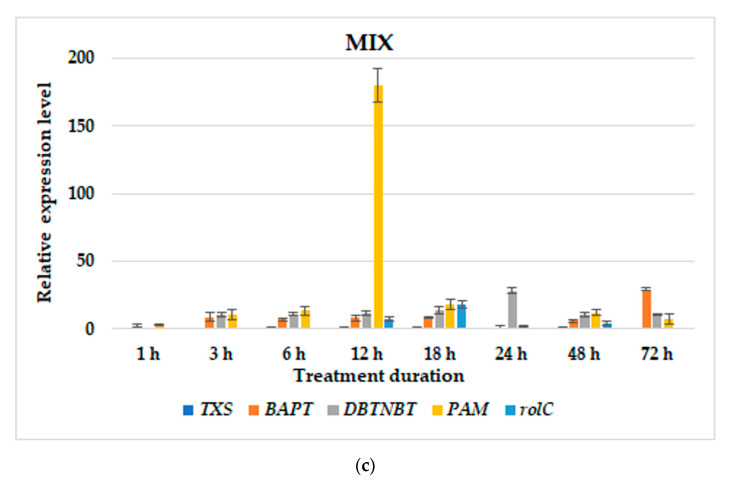
The effect of elicitation on relative gene expression profiles in hairy root cultures treated with ETOH (**a**), MeJA(**b**) or MIX (**c**). Data represent mean values ± SD from three independent experiments.

In culture variants without BABA, ETOH added individually caused an increased in steady-state transcript levels. The most affected were *TXS*, *DBTNBT* and *rolC* after 24 h, and *PAM*, which peaked twice after 3 and 24 h (Figure 4a). However, these inductions of gene expression were not translated into paclitaxel production (Figure 2a). MeJA elicitation caused a rise in the gene expression levels of almost all genes investigated, except *TXS*. The coincidence in the highest transcript abundance was observed in the expression levels of *PAM* and *rolC*. *BAPT* peaked after 12 h of treatment, although the maximum expression was noted after 72 h. Meanwhile, the maximum level of *DBTNBT* expression was detected at 24 h post-elicitation (Figure 4b). These enhancements in transcript abundance reflected the capacity in paclitaxel accumulation, which was the highest among these culture variants (Figure 2a). In the hairy roots elicited with MIX, the significant induction of late-pathway genes was determined with the highest *PAM* transcript abundance 12 h after the addition of elicitors (Figure 4c). The expression of *rolC* was also remarkably affected; however, its levels were over 2-fold lower than under MeJA added as a single elicitor.

**Figure 5:**
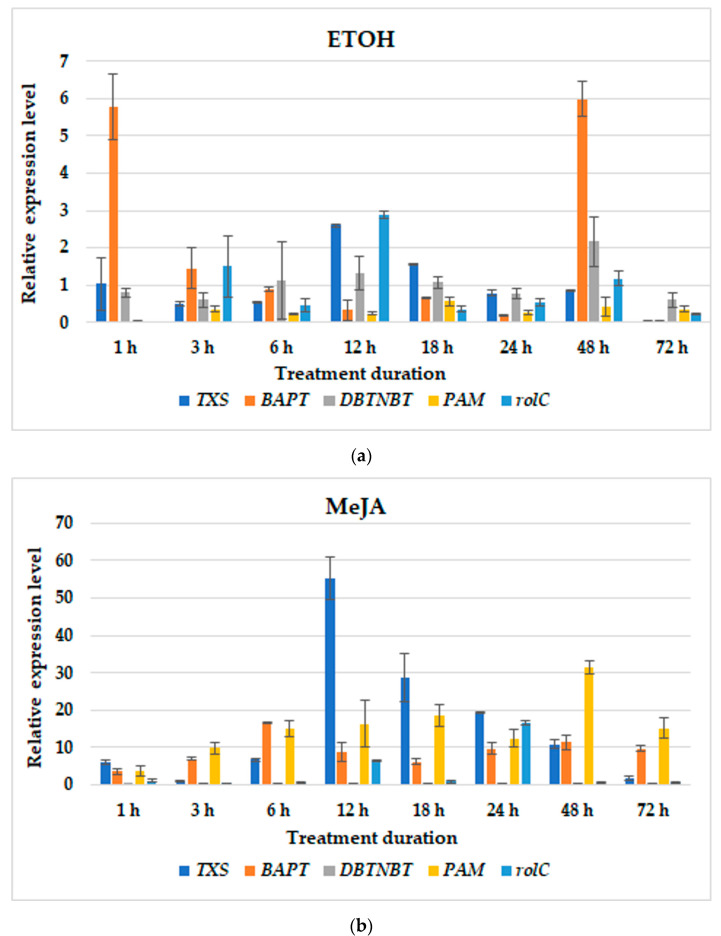

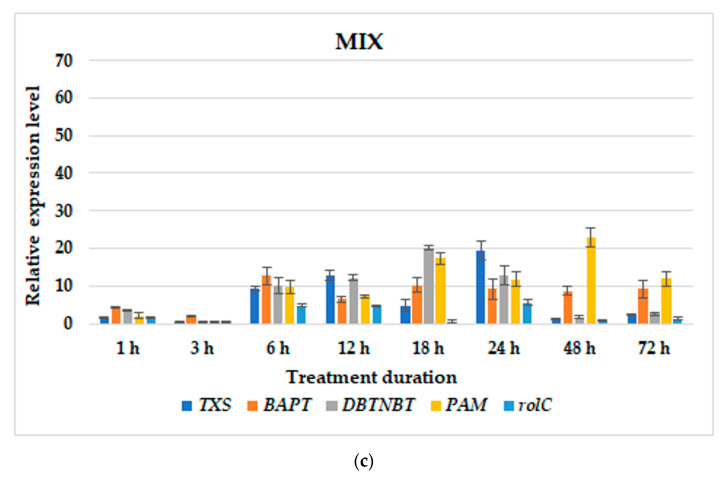
The effect of BABA24 treatment followed by ETOH (**a**), MeJA (**b**) or MIX (**c**). Elicitation on relative gene expression profiles in hairy root cultures. Data represent mean values ± SD from three independent experiments.

After BABA24 treatment followed by ETOH, the most induced gene was *BAPT*, which peaked two times, i.e., after 1 and 48 h (Figure 5a). The paclitaxel production under this treatment was the highest after 24 h, followed by its decline. After the addition of MeJA to BABA24 treated hairy roots, the transcript abundance was higher than in the BABA24E variant, except the *DBTNBT* gene. *TXS* demonstrated the highest gene expression levels, with a peak after 12 h (Figure 5b). Under these conditions, paclitaxel accumulation was observed starting after 72 h of elicitation, with its maximum after 7 days, and was over 3-fold higher than in the BABA24E variant. In the BABA24MIX and BABA24MeJA variants, a similar pattern of gene expression was observed (Figure 5c). However, in opposition to the BABA24MeJA variant, the *DBTNBT* gene were considerably higher expressed. Nevertheless, this was not reflected in the yield of paclitaxel, which was higher than in the BABA24MeJA variant, but this difference was not statistically significant (*p* < 0.05). Further, the shift in the time required to achieve this compound maximum concentration was noted, from 7 days to 14 days in BABA24MeJA and BABA24MIX variants, respectively.

**Figure 6:**
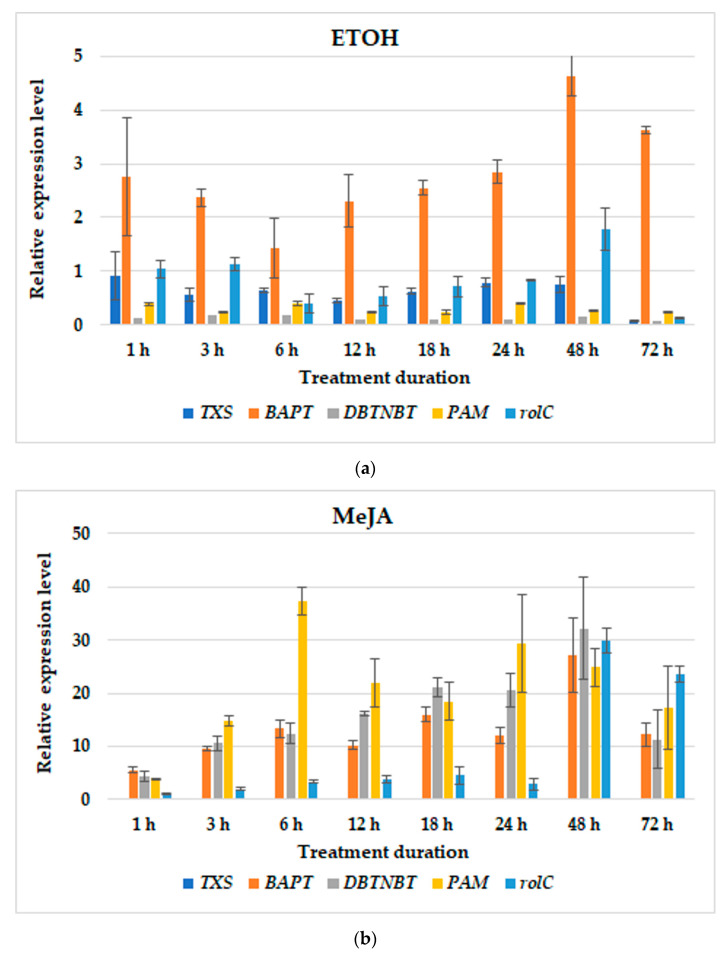

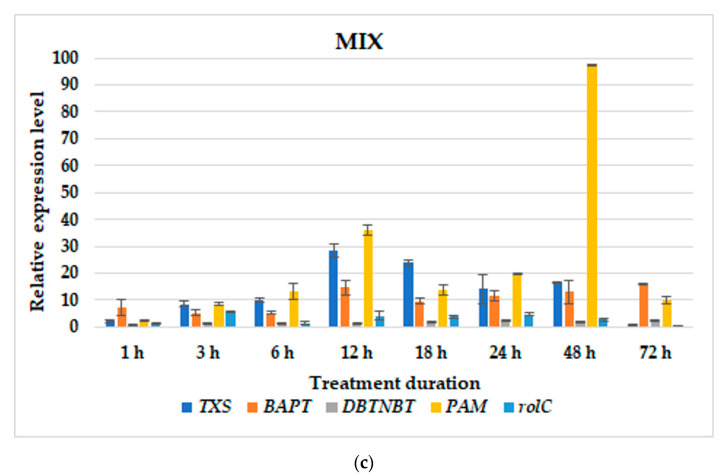
The effect of BABA1 treatment followed by ETOH (**a**), MeJA (**b**) or MIX (**c**) elicitation on relative gene expression profiles in hairy root cultures. Data represent mean values ± SD from three independent experiments.

In the BABA1E variant, within the first hour the enhancement in steady-state transcript levels of almost all examined genes except *DBTNBT* (Figure 6a) was noted; however, this rise was not translated into paclitaxel production. In the BABA1MeJA variant, starting from the first hour, an increased expression of late-pathway *BAPT*, *DBTNTB* and *PAM* genes, as well the *rolC* gene, was observed with continued abundance extending through to 72 h (Figure 6b). The most inducible gene was *PAM*, which peaked after 6 h of elicitation. *TXS* expression, an early-pathway gene, was very low, lower even than in the BABA1E variant. Eventually, under these conditions, paclitaxel production was detected at 7 and 14 days after elicitor feeding. In the BABA1MIX variant, the *TXS*, *BAPT* and *PAM* abundance of transcript was elevated in comparison to previous BABA1 variants, especially *PAM*, which peaked after 12 h of treatment (Figure 6c). It should be noticed that *TXS* transcript levels were the highest among all BABA1 variants. *DBTNTB* was also induced; however, to a remarkably lesser extent than in the BABA1MeJA variant. The *rolC* gene expression was also lower than in the BABA1MeJA variant, but higher than in the BABA1E variant (Figure 6). Taken all together, paclitaxel yield determined in this variant was the highest among all tested variants, with its concentration being detected starting from 48 h post-treatment and reaching its maximum after 14 days.

## 4. Discussion

The concept of harnessing the primed response induction to enhance secondary metabolite production in plant cell in vitro cultures has not been investigated to date. However, there are some reports describing the physiological effects of priming in the aspect of phytoalexin production and secretion in reaction to priming. Until now, it was reported that the biosynthesis of various classes of secondary metabolites, i.e., phenolic compounds, glucosinolates and volatile compounds, was induced, and that their profile depended on plant species and priming inducer [22].

The current study aimed to develop a new elicitation strategy involving the primed defence response approach, combined with subsequent elicitor treatment. It assumes significant improvements in the yield of paclitaxel as a result of the preconditioning of *T*. × *media* hairy root cultures with BABA for enhanced response, combined with subsequent elicitor (MeJA) or elicitors (MIX) treatment.

Among the many biotechnological approaches developed to improve paclitaxel and other taxane production in *Taxus* spp. in vitro cultures, the most effective seemed to be elicitation with MeJA [5]. Numerous investigations were undertaken to elucidate the effect of MeJA on taxane biosynthesis on a molecular level. The 1–7-day delay was reported between a time course for mRNA accumulation of the known taxane biosynthetic genes and taxane production [15,23] and upregulation, not only of genes involved in taxane biosynthesis, but also genes engaged in the stimulation of plant hormone and phenylpropanoid biosynthesis, MeJA signalling, taxane transport and degradation, as well as transcriptional regulation [23,24,25,26,27]. The taxane biosynthetic pathway is not fully characterised; according to current knowledge, it requires approximately 20 enzymatic steps [5] and possible rate-limiting steps were indicated in the late pathway, i.e., those controlled by *BAPT* and *DBTNBT*.

In previous studies dealing with *T*. × *media* hairy root cultures, biomass retardation upon MeJA or MIX addition was reported [12,13,17,20,28,29]. In the present study, root growth was not negatively affected by culture conditions, and further significant (*p* < 0.05) biomass increase was noted in cultures supplemented with BABA alone. In the previous study on hairy root cultures of *S*. *miltiorrhiza*, it was described that BABA added at higher dosages (1 mM) resulted in slight root growth suppression, although this effect was not observed when BABA was used in combination with MeJA, which is in accordance with the results of the present study [11]. In the other study, it was demonstrated that BABA stimulated the photosynthesis and growth of *Medicago interexta* sprouts, to a higher extent when applied together with selenium nanoparticles (SeNPs) [30].

Until now, the priming strategy combined with elicitation has not been applied nor analysed in relation to enhancing paclitaxel biosynthesis. The results of the present study have been compared with previous investigations reporting changes in gene expression involved in taxane biosynthesis (Table 1).

BABA application caused the induction of investigated gene expression, even over their levels in control; however, it was not connected with the induction of paclitaxel biosynthesis. This could be attributed to the possible effect of BABA on changes in DNA methylation pattern which was postulated to affect taxane production in vitro [34,35,36].

Paclitaxel production was observed when the medium was supplemented with elicitor/s (Figure 2a). If the influence of ETOH on paclitaxel accumulation was taken into consideration, it was revealed that its content was detected only in the BABA24E variant, and in low quantities (Figure 2b), so it could be assumed that the effect of ETOH on paclitaxel biosynthesis could be irrelevant. In variants supplemented with BABA, it was demonstrated that the duration of preconditioning had a significant impact on paclitaxel productivity. The highest paclitaxel concentration was detected after a two-week elicitation in BABA1MIX variants, which corresponded to the highest expression levels of *TXS* and *BAPT* genes. *DBTNBT*, *PAM* and *rolC* transcript abundance was higher in the BABA1MeJA variant, in which *BAPT* expression was on a parallel level with the BABA1MIX variant, while *TXS* expression was very low (Figure 5 and Figure 6). The activity of the *TXS* gene, committing the first step of taxane biosynthesis, was claimed not to be rate-limiting on taxane biosynthesis [15,16], which also found confirmation under the conditions of the present study. The results of the current investigations seem to confirm that among all investigated genes herein, *BAPT* could play an essential role in taxane productivity (which was previously postulated [15,16,34]), but also, *PAM* upregulation could affect paclitaxel biosynthesis capacity.

The maximum paclitaxel yield in the present study was determined in the BABA1MIX variant; it was also the highest paclitaxel productivity reported for this hairy root line [37]. Previously, the importance of preincubation with BABA on the tanshinone total yield was described in *S*. *miltiorrhiza* hairy root cultures, and the authors pointed out that tanshinone productivity increased steadily with the prolongation of BABA treatment [38]. Further, it was demonstrated that BABA influenced results, especially in combination with SeNPs’ potentiated accumulation of phenolics and flavonoids, and especially when they were applied together [30]. Next, the stronger activation of defence response was observed in BABA-primed mango fruit to subsequent inoculation with *Colletotrichum gloeosporoides* [39]. Upon treatment with only BABA, in comparison to control, the total content of phenolics, flavonoids and lignin was significantly higher, which is in opposition to the results of the present study. On the other hand, the BABA pretreatment followed by *C*. *gloeosporoides* inoculation caused an acceleration in their increase and accumulation, which corroborates the present findings.

In plant secondary metabolism, the stimulating function of *ROL* genes was reported, as well as the effect of single *ROL* gene relays on plant species and groups of secondary metabolites [40,41]; however, until now it was not examined in *Taxus* spp. hairy roots. In the present study, the *rolC* gene was highly expressed under MeJA elicitation and in the BABA1MeJA variant, which corresponds to a high concentration of paclitaxel under those conditions. Nevertheless, despite high amounts of paclitaxel detected in these variants, its yield was the highest in BABA1MIX, i.e., the treatment where a noticeable *rolC* expression was also correlated with the upregulation of the other investigated genes (Figure 2 and Figure 6).

## 5. Conclusions

In *T*. × *media* KT hairy root line cultures, pretreatment with BABA for one week followed by elicitation with a mixture of MeJA, sodium nitroprusside and L-phenylalanine was proved to be the most effective in the stimulation of paclitaxel biosynthesis. This coincided with considerably higher *TXS*, *BAPT* and *PAM* gene expression levels among all investigated genes, which could indicate that the orchestration of gene expression of early and late biosynthesis steps is indispensable for enhanced paclitaxel biosynthesis. The duration of BABA pretreatment seems to have the most pronounced impact on its productivity. The yield of paclitaxel reported in the present study is the highest achieved in *T*. × *media* hairy root cultures to date.

Although the knowledge on the regulation of the paclitaxel biosynthesis pathway in the KT hairy root line was broadened, further investigation is necessary to identify its controlling and limiting steps, and on the levels of transcription factor activity, as well as DNA methylation.

## Figures and Tables

**Table 1:** The effect of culture conditions on changes in taxane biosynthesis gene expression profile.

*Taxus* Spp.	Type of Culture	Elicitor/s	Peak in Gene Expression	Reference
*T*. *cuspidata*	cell suspension	MeJA	*TXS*–18 h; *BAPT*–6 h; *DBTNBT* and *PAM*–6 h till	[15]
*T*. × *media*	cell suspension; two lines: TXS—carrying *TXS* transgene and *ROL* genes of *A*. *rhizogenes;* ROLC—with *ROL* genes of *A*. *rhizogenes*	MeJA	*TXS:* TXS line–12 h; ROLC line–48 h	[31]
*T*. *baccata*	cell suspension	MeJA; vanadyl sulphate (VS)	MeJA: *TXS*–24 h; *BAPT*–12h and at day 20;VS-*TXS*–8 day; *BAPT*–at 4 day;MeJA and VS: *TXS*–4 h; *BAPT*–12h	[32]
*T*. × *media*	cell suspension; line TXS line-carrying *TXS* transgene	MeJA; coronatine (COR)	MeJA: *TXS*–24 h; *BAPT* and *DBTNBT*–4 day; *PAM*–1h and 4 day;COR: *TXS*–24 h; *BAPT*–24 h; *DBTNBT*–48 h; *PAM*–48 h	[33]
*T*. × *media*	cell suspension; line TXS line-carrying *TXS* transgene	MeJA, randomly methylated-β-cyclodextrin (M-β-CD) separately or combined	MeJA: *TXS*–72 h; *BAPT*–96 h; *DBTNBT*–72 h; M-β-CD: *TXS*–72 h; *BAPT*–1-4 h; *DBTNBT*–72 h;MeJA+M-β-CD: *TXS*–72 h; *BAPT*–4 h; *DBTNBT*–72 h;	[18]
*T*. × *media*	two lines of hairy roots: KT and ATMA	MeJA, sodium nitroprusside, L-phenylalanine, degassed perfluorodecalin additional sucrose	KT line: *TXS*–12 h; *BAPT*–6 h and at parallel level till 24 h; *DBTNBT*–12 h and 48 h;ATMA line: *TXS*–48 h; *BAPT*–48 h; *DBTNBT*–6 h and 48 h;	[17]
*T*. × *media*	hairy roots of line KT	BABA; MeJA; MIX; BABA24+MeJA or MIX; BABA1+MeJA or MIX	BABA: *TXS*–1 h and 48 h; *BAPT*–6 h, at 5 and 10 day; *DBTNBT*–at 5 day; *PAM*–24 h; *rolC*–24 h;MeJA: *TXS*–72 h; *BAPT*–72 h; *DBTNBT*–24 h; *PAM*–12 h; *rolC*–12 h;MIX: *TXS*–18 h; *BAPT*–72 h; *DBTNBT*–24 h; *PAM*–12 h; *rolC*–18 h;BABA24MeJA-*TXS*–12 h; *BAPT*–6 h and 48 h; *DBTNBT*–24 h; *PAM*–48 h; *rolC*–24 h;BABA24MIX-*TXS*–12 h and 24 h; *BAPT*–6 and at parallel level till 72 h; *DBTNBT*–6 h and at parallel level till 24 h; *PAM*–48 h; *rolC*–24 h;BABA1MeJA: *TXS*–48 h; *BAPT*–48 h; *DBTNBT*–48 h; *PAM*–6 h; *rolC*–48 h;BABA1MIX: *TXS*–12 h; *BAPT*–12 h; *DBTNBT*–48 h; *PAM*–48 h; *rolC*–3 h;	present study

## Data Availability

Not applicable.

## Supplementary Materials

The following are available online at https://www.mdpi.com/article/10.3390/cells11132062/s1. Figure S1: The geNorm ranking of candidate reference genes *18S rRNA*, *TBC41*, *TUBB* analysed using the tool RefFinder and agarose gel.; Table S1: Characteristics of primers used in qPCR reaction.
