# Supplementary material for: Application of Priming Strategy for Enhanced Paclitaxel Biosynthesis in Taxus × Media Hairy Root Cultures
Source: Cells. 2022 Jun 29;11(13):2062. doi: 10.3390/cells11132062 (PMC9265826; doi:10.3390/cells11132062)
Supplement: Supplementary file 1 [file cells-11-02062-s001.zip › FigureS1.pdf]

Application of priming strategy for enhanced paclitaxel biosynthesis in *Taxus* x *media* hairy root cultures

Katarzyna Sykłowska-Baranek[1], Grażyna Sygitowicz[2*], Agata Maciejak-Jastrzębska[2], Agnieszka Pietrosiuk[1] and Anna Szakiel[3]

[1] Department of Pharmaceutical Biology and Medicinal Plant Biotechnology, Faculty of Pharmacy, Medical University of Warsaw, 1 Banacha Str., 02-097 Warsaw, Poland; katarzyna.syklowska-baranek@wum.edu.pl (K.S.B.); agnieszka.pietrosiuk@wum.edu.pl (A.P.)

[2] Department of Clinical Chemistry and Laboratory Diagnostics, Medical University of Warsaw, 1 Banacha Str., 02-097 Warsaw, Poland; gsygitowicz@poczta.onet.pl (G.S.); agata.maciejak@wum.edu.pl (A.M-J.)

[3] Department of Plant Biochemistry, Faculty of Biology, University of Warsaw, 1 Miecznikowa Str., 02-096 Warszawa, Poland; szakal@biol.uw.edu.pl (A.S.)

* Correspondence: gsygitowicz@poczta.onet.pl (G.S.)

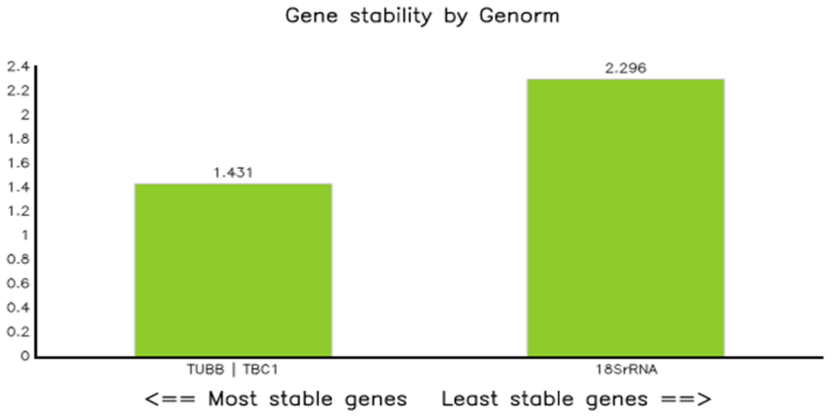

(**a**)

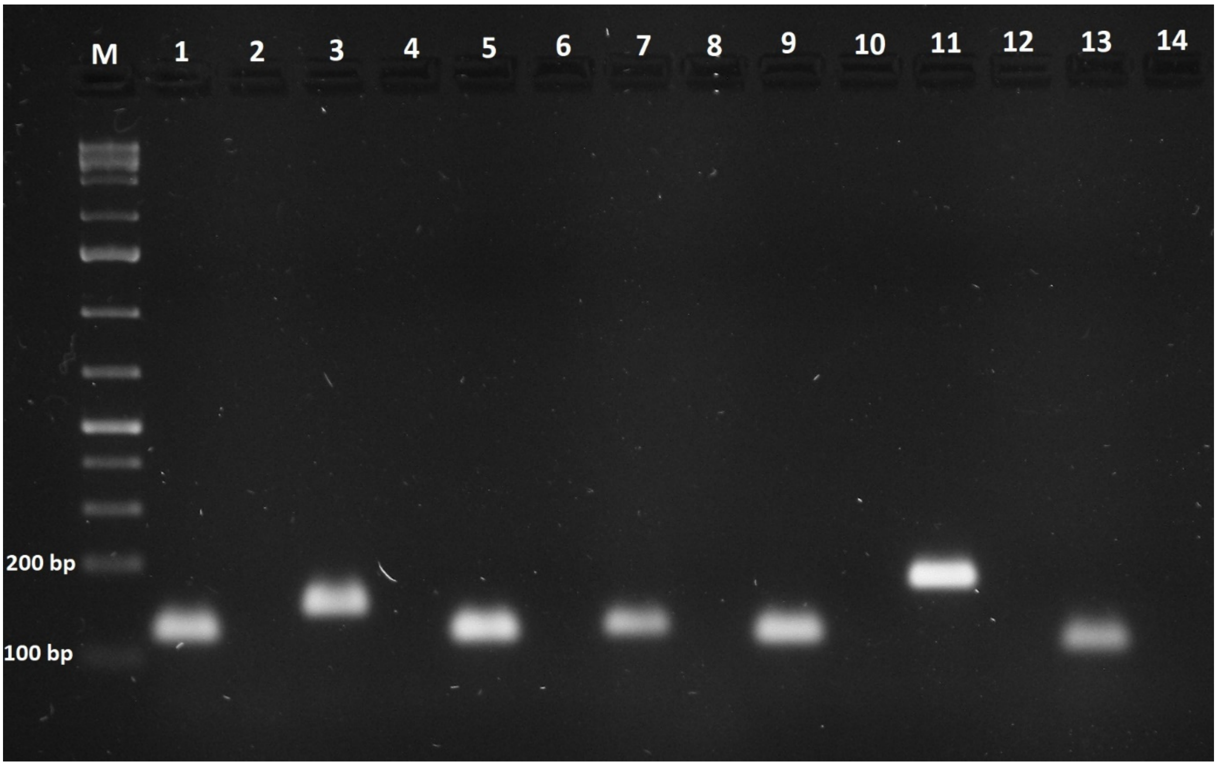

(**b**)

Figure S1. (**a**) The geNorm ranking of candidate reference genes *18S rRNA*, *TBC41*, *TUBB* analyzed using the tool RefFinder (https://heartcure.com.au); (**b**) Agarose gel (2%) electrophoresis of the qPCR products.

Lane M – 100 bp ladder; lanes 1, 2 – *BAPT* positive and negative controls, respectively; lanes 3, 4 – *DBTNBT* positive and negative controls, respectively; lanes 5, 6 – *18S rRNA* positive and negative controls, respectively; lanes 7, 8 – *PAM* positive and negative controls, respectively; lanes 9, 10 – *TBC41* positive and negative controls, respectively; lanes 11, 12 – *TUBB* positive and negative controls, respectively; lanes 13, 14 – *TXS* positive and negative controls, respectively.
