# Supplementary material for: Application of Priming Strategy for Enhanced Paclitaxel Biosynthesis in Taxus × Media Hairy Root Cultures
Source: Cells. 2022 Jun 29;11(13):2062. doi: 10.3390/cells11132062 (PMC9265826; doi:10.3390/cells11132062)
Supplement: Supplementary file 1 [file cells-11-02062-s001.zip › Table S1.pdf]

Application of priming strategy for enhanced paclitaxel biosynthesis in *Taxus* x *media* hairy root cultures

Katarzyna Sykłowska-Baranek[1], Grażyna Sygitowicz[2*], Agata Maciejak-Jastrzębska[2], Agnieszka Pietrosiuk[1] and Anna Szakiel[3]

[1] Department of Pharmaceutical Biology and Medicinal Plant Biotechnology, Faculty of Pharmacy, Medical University of Warsaw, 1 Banacha Str., 02-097 Warsaw, Poland; katarzyna.syklowska-baranek@wum.edu.pl (K.S.B.); agnieszka.pietrosiuk@wum.edu.pl (A.P.)

[2] Department of Clinical Chemistry and Laboratory Diagnostics, Medical University of Warsaw, 1 Banacha Str., 02-097 Warsaw, Poland; gsygitowicz@poczta.onet.pl (G.S.); agata.maciejak@wum.edu.pl (A.M-J.)

[3] Department of Plant Biochemistry, Faculty of Biology, University of Warsaw, 1 Miecznikowa Str., 02-096 Warszawa, Poland; szakal@biol.uw.edu.pl (A.S.)

* Correspondence: gsygitowicz@poczta.onet.pl (G.S.)

Table S1. Characteristics of primers used in qPCR reaction.

Gene symbol	Gene name	GenBank accession no.	Sequence of primer	Amplicon length (bp)	Efficiency (%)	Reference
BAPT	Baccatin III: 3-amino, 3-phenylpropanoyltransferase	AY563630.1	F: 5′CAGGTTCGTTAGCAGAGTTCCA3′<br>R: 5′ATGTTGTCAATGGCGGAGAGA3′	106	94	[2]
DBTNBT	3′-N-debenzoyl-2′-deoxytaxol-N-benzoyltransferase	AY563629.1	F: 5′CGATGCGTCCACCTCCAATAG3′<br>R: 5′CCAATGCTGCTACGACCTCAA3′	141	85	[2]
PAM	Phenylalanine aminomutase	AY866411.1	F: 5′CCCGGAGGCATGACGTGAAG3′<br>R: 5′CGCCGTCTTCCGCCTTGC3′	99	96	[1]
rolC	Cytokinin-β-glucosidase	MT514512.1	F: 5′CTGTACCTCTACGTCGACT3′<br>R: 5′AAACTTGCACTCGCCATGCC3′	351		
TBC41	3,5-epimerase-4-reductase-like protein	MN173618.1	F: 5′CAAGAAGAAAGAGTCAGCAAATGG3′<br>R: 5′GGAACGACATGACATTATGAATAGC3′	91	70	[1]
18S rRNA	18S ribosomal RNA	AY544989.1	F: 5′GTGACGGGTGACGGAGAATTAG3′<br>R: 5′CGTGAGCCCAGTATTGTTATTTATTGTC3′	144		[2]
TUBB	β-Tubulin	AB918700.1	F: 5′GGCTTTCTTGCACTGGTACAC3′<br>R: 5′CATCTCCTGAAACTACCGACTC3′	164	92	[3]
TXS	Taxadiene synthase	AY461450.2	F: 5′TGCGTGCCCTGTATGTATTCC3′<br>R: 5′GACCGATTCCGAGATGCTCAAT3′	79	115	[3]

1. Sabater-Jara, A. B.; Onrubia, M.; Moyano, E.; Bonfill, M.; Palazón, J.; Pedreño, M. A.; Cusidó, R. M. Synergistic effect of cyclodextrins and methyl jasmonate on taxane production in *Taxus x media* cell cultures. *Plant Biotechnol. J.* **2014**, *12*, 1075–1084, doi:10.1111/pbi.12214.
2. Onrubia, M.; Moyano, E.; Bonfill, M.; Cusidó, R. M.; Goossens, A.; Palazón, J. Coronatine, a more powerful elicitor for inducing taxane biosynthesis in *Taxus media* cell cultures than methyl jasmonate. *J. Plant Physiol.* **2013**, *170*, 211–219, doi:10.1016/j.jplph.2012.09.004.
3. Sykłowska-Baranek, K.; Rymaszewski, W.; Gaweł, M.; Rokicki, P.; Pilarek, M.; Grech-Baran, M.; Hennig, J.; Pietrosiuk, A. Comparison of elicitor-based effects on metabolic responses of *Taxus × media* hairy roots in perfluorodecalin-supported two-phase culture system. *Plant Cell Rep.* **2019**, *38*, 85–99, doi:10.1007/s00299-018-2351-0.
