# Supplementary figures and images for: Application of Priming Strategy for Enhanced Paclitaxel Biosynthesis in Taxus × Media Hairy Root Cultures
Source: Cells. 2022 Jun 29;11(13):2062. doi: 10.3390/cells11132062 (PMC9265826; doi:10.3390/cells11132062)

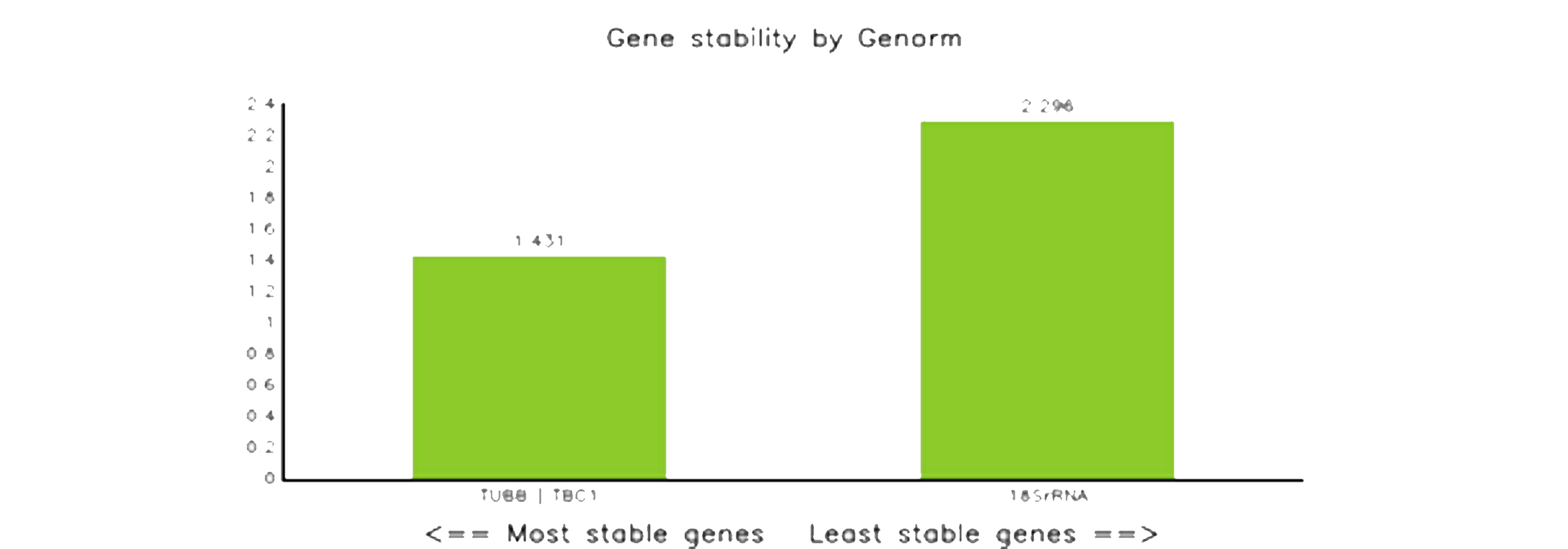

Supplement: Supplementary file 1 [file cells-11-02062-s001.zip › Figure S1a.tif]

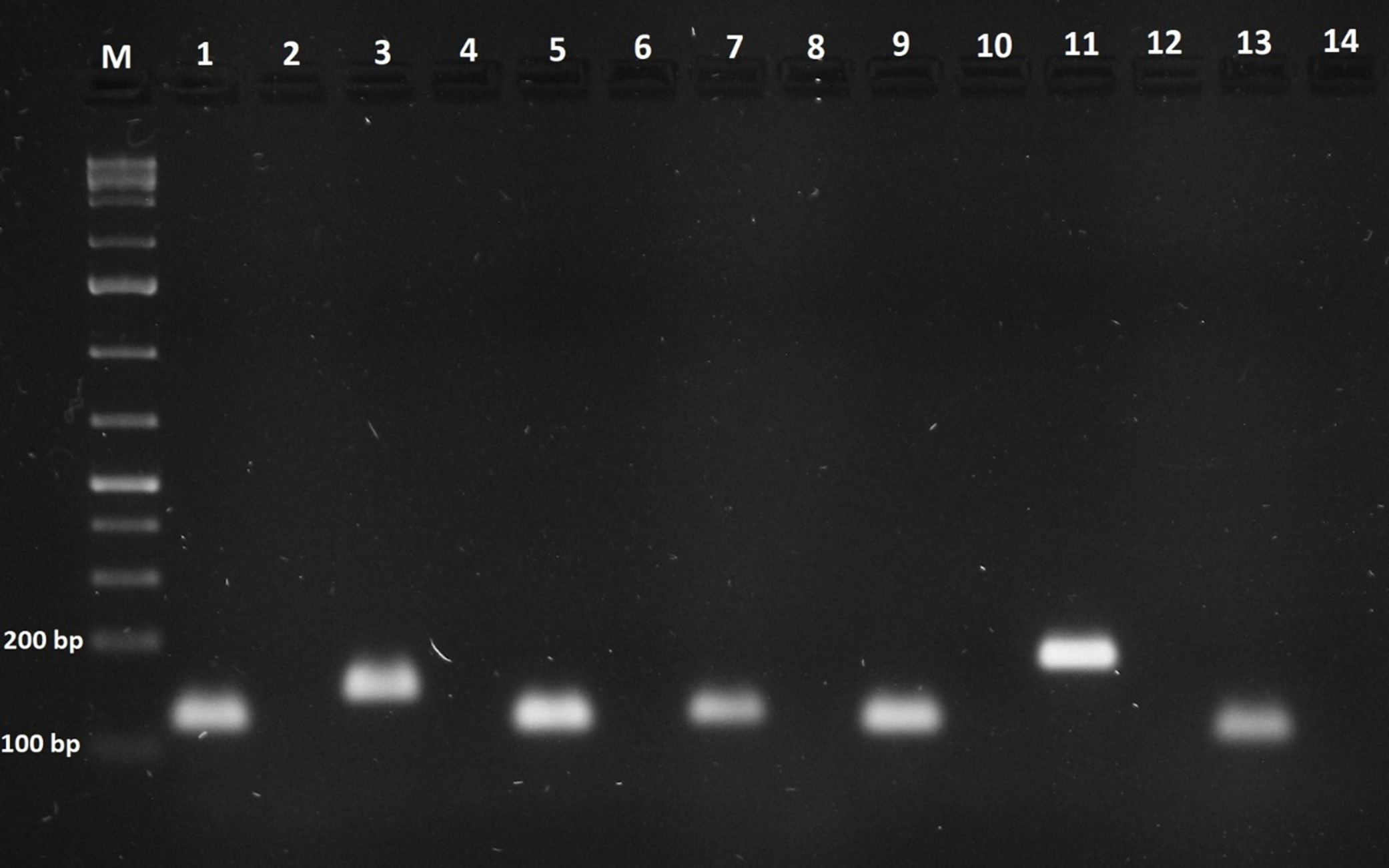

Supplement: Supplementary file 1 [file cells-11-02062-s001.zip › Figure S1b.tif]
